# The grand challenges in obstetric and pediatric pharmacology

**DOI:** 10.3389/fphar.2013.00170

**Published:** 2013-12-30

**Authors:** Anne Zajicek, Jeffrey S. Barrett

**Affiliations:** ^1^Eunice Kennedy Shriver National Institute of Child Health and Human Development, National Institutes of HealthBethesda, MD, USA; ^2^Interdisciplinary Pharmacometrics Program, Pediatric Clinical PharmacologySanofi, Philadelphia, PA, USA

**Keywords:** obstetrics, pediatrics, pharmacology, medications, therapeutics

Obstetric and pediatric pharmacology urgently need creative, collaborative, multidisciplinary research approaches to optimize medication use in these vulnerable patient populations. Optimal use of medications in these populations, in order to provide greater understanding and confidence for both patients and caregivers, and training the next generation of pediatric and obstetric pharmacologists, are our primary goals.

## Background

Medication use in children and pregnant women is generally empiric, based on data derived from the (non-pregnant) adult population. Assumptions from the adult population are embedded into dosing, efficacy and safety expectations for these populations. Drugs are almost always developed for adult patients given the economics that drive the pharmaceutical industry. The potential for consideration of use in children for the same adult indication occurs later in the drug development process (http://www.gpo.gov/fdsys/pkg/BILLS-112s3187enr/pdf/BILLS-112s3187enr.pdf). Consideration of indications that exist only in neonates or during pregnancy (e.g., necrotizing enterocolitis, respiratory distress syndrome, preterm labor, pre-eclampsia) have enormous long-term health consequences (Calkins and Devaskar, 2011) but yet there is little in the way of approved medications for any of these conditions, and a paucity of research in determining the mechanisms and potential drug targets for these indications. Both settings provide significant challenge for research and development based on the vulnerability, size and complexity of these populations.

Traditional thought has been that children and pregnant women are healthy and do not take medications. This is not the case. Twelve percent of children in the US have asthma, and most children at one time or another have otitis media, to name two common pediatric conditions. There are approximately 12,000 new pediatric cancer diagnoses a year, using complex cancer chemotherapy regimens with medications developed decades ago. For pregnant women, six is the average number of medications taken during a pregnancy (Lee et al., 2006). Given the later age of pregnancies, and increase in prevalence in the US, more women have chronic health conditions such as Type 2 diabetes mellitus, hypertension, and depression. There are also pregnancy-related conditions such as gestational diabetes (or undiagnosed Type 2 diabetes mellitus), pre-eclampsia, and pre-term labor, which although self-limited can have life-long consequences for the mother and child. Prevention and treatment of these conditions therefore would have long-term benefits for both mother and child.

## Innovation

Medical treatment of fetal health conditions is an application of both pediatric and obstetric pharmacology. Two specific examples are in the prevention of respiratory distress syndrome by prenatally administered progesterone to the pregnant woman in preterm labor, and treatment of fetal supraventricular tachycardia (SVT). The various complexities involved, including diagnosis of fetal SVT, choice of drug and dose, the various pharmacokinetic compartments including the placenta illustrate some of the challenges of developmental pharmacology (Tavera et al., 2010).

New drug development which is informed by fetal drug targets would potentially prevent undesired off-target toxicity, while maximizing benefit. The other side of fetal effects is fetal toxicity including fetal malformations. Determination of developmental targets, and on- and off-target effects from drugs, would be highly desirable, in order to develop new drugs or repurpose old ones which would be safe for the obstetric population and the developing fetus. This would be further improved with mechanistic-based pre-clinical models, as the precise mechanisms of drug effects could be identified (Acosta et al., 2013).

Concerns resulting from unwanted drug effects have weighted the risk: benefit ratio for new drug development in a more conservative manner to the detriment of these populations. Obstetrics continues to lag behind. Fisk and Atun (2008) demonstrate this point in a recent publication. Looking at new drug development from 1980 to 2007, 17 drugs were under development for obstetric indications, 660 for cardiovascular disease (a high-prevalence indication), and 34 for amyotrophic lateral sclerosis (ALS), which is an orphan indication. To put this in the proper context, there are approximately 4 million live births in the US, and with 6–10% complicated by pre-eclampsia and 12% with pre-term birth; the incidence of ALS is 2:100,000.

Preclinical models have been beneficial for the understanding of drug actions in vulnerable populations. Extrapolation from these primarily descriptive models into humans has been problematic and sometimes contradicted by human experience. Explanations include the lack of a precise mechanism of drug action, whether beneficial or harmful; different receptors, transporters, or other drug targets in pre-clinical models; differences in placentation and placental perfusion between species; lack of age correlation between humans and juvenile animal models; and lack of precise phenotyping. We remain hopeful that investigation of developmental and mechanistic models of drug effect will provide more accurately predicted human response and specifically obstetric and pediatric pharmacology knowledge.

Development of drug targets for is difficult but necessary for pediatric and obstetric new drug development. Given the dynamic nature of these populations, signaling pathways, receptors and transporters are in constant development and maturational flux. Attempts must be made to determine “druggable” pathways or targets to treat or prevent disease in children and during pregnancy. The National Institutes of Health (NIH) is involved in developing such targets under its drug repurposing activities at the National Center for Advancing Translational Sciences (http://www.ncats.nih.gov/).

## Translating research milestones to pharmacotherapeutic outcomes

Consistency and agreement in phenotyping and in clinical trial endpoints is a necessary but difficult concept. Difficulties in determining efficacy and safety of medications across clinical trials using meta-analyses stem from this very problem- lack of uniformity in nomenclature and data definitions though the necessity of developing adequate methodologies is also critical. Phenotyping is inherently difficult in children and pregnant women given the developmental nature childhood and pregnancy. Carleton (Shaw et al., 2013) has done a clever job of connecting pharmacogenomics with drug safety by using a statistical approach to candidate genes coupled with consistent phenotyping. Likewise, an understanding of clinically relevant sources of variation in PK and drug response to optimize dosing: integration of pharmacogenomics and pharmacometric approaches would be desirable.

Creativity and innovation are required to develop informative pharmacology studies, and to make maximum use of biological samples and clinical data. Clinical data gathering, whether through the use of electronic medical records, paper records or registries, and biological sample gathering, using scavenged samples, as well as other novel methods, would be highly valuable to potentially determine subpopulations of responders and non-responders and development of potential novel drug targets. Alternative clinical trial designs, registry trials and observational designs to improve the recruitment and retention of study subjects are highly applicable to children and pregnant women (Shields and Lyerly, 2013).

Biomarkers are needed for pediatric and obstetric drug development. There is a paucity of biomarkers for either population. The necessity for extended follow-up of pediatric and obstetric populations to ensure safety is a primary reason for the lack of drug development in these populations. Development and validation of biomarkers and surrogate markers is crucial. Again, there is a need to understand disease processes and on- and off-target drug effects in order for biomarkers to be discovered and validated.

Pharmacoepidemiology in pediatric and obstetrics is critical for defining exactly what medications patients are taking and for what conditions. This has been problematic: medications and indications have not been generally linked in claims databases, so it has been quite difficult to determine the condition that the medication is being used to treat. Pharmacoepidemiology can provide the requisite data granularity regarding what populations, and subpopulations including age and geographic area, are being prescribed and for what indication(s) as well as adverse events and drug-drug interactions associated with medication use. Coupling pharmacoepidemiologic knowledge with relationships that define pediatric disease progression and drug behavior in children provide a powerful venue to evaluate the likelihood of clinical success of new medicines in these target populations.

Medical informatics as a discipline has yielded success in the improvement of therapeutics selection and use and is being incorporated into medical practices throughout the US while having been standard in many parts of the world. Informing such approaches requires translational scientists to understand both disease and population more intimately than in the past. While IT hurdles continue to exist, these would seem to be more surmountable given the investment from various sources (e.g., hospital information technology, electronic health records, vendors, new legislation and incentives, third party payers). A major utility of new informatics databases is, of course, to improve patient care. Understanding the relationship of medication use to adverse events in children and pregnant women, as well as birth outcomes, would be an obvious benefit (Etwel et al., 2013). Clinical investigation of current practices, in comparison to presumed best practices, should be possible and encouraged.

Data bridges between basic, translational and clinical sciences are needed to fill gaps in knowledge regarding mechanistic rationale for appropriate medication use. A mechanistic understanding of drug disposition and pharmacologic effects, including adverse reactions related to drug therapy, must begin at a basic science level. Identification of current drug targets and development of new ones, both in the fetus, child, and pregnant woman, are all critical. Incorporation of pharmacogenetics/genomics approaches to develop relationships between drug administration and drug effect (efficacy/toxicity) represents a new frontier for the development of more targeted approaches to increase efficacy and reduce toxicity.

## Conclusions

The adult clinical development paradigm involves sequential, discrete preclinical and clinical investigation that ultimately drives decision making based on achieving (or not) development milestones. In contrast, the pediatric setting for drug development is typically defined by bridging criteria which is strongly influenced by the adult evidence for safety and efficacy. Recent US and EU pediatric legislation has caused a shift in this timeline, moving drug testing for children earlier in the drug development process. The formation of pediatric-centered research groups within pharma is some indication that resources for pediatric drug development are more appreciated now than in the past.

The intent of this Journal is to serve as the voice for the community and also these understudied and underserved populations. We hope that this new journal will provide a venue for new methodologies for data analysis, and alternate clinical trial designs for small populations. By encouraging the submission of publications proposing the development of innovative study designs and tools to evaluate medications in these populations, we endeavor to move this field of investigation further. As pharma is developing new drugs for specific pharmacogenetic subtypes, the conduct of clinical trials, including use of alternative clinical trial designs, to accommodate small populations could not be timelier.

There are large knowledge gaps, which need to be filled in order to best treat children and pregnant women. We look forward to developing a creative, interactive publication resource for members of the pediatric and obstetric research communities in order to improve their health.

## Disclaimer

Comments and views of the authors do not necessarily represent the views of the NICHD.
